# Efficacy of mesenchymal stem cell therapy in systolic heart failure: a systematic review and meta-analysis

**DOI:** 10.1186/s13287-019-1258-1

**Published:** 2019-05-31

**Authors:** Mengkang Fan, Yin Huang, Zhangwei Chen, Yan Xia, Ao Chen, Danbo Lu, Yuan Wu, Ning Zhang, Juying Qian, Junbo Ge

**Affiliations:** 1 Department of Cardiology, Shanghai Institute of Cardiovascular Diseases, Zhongshan Hospital, Fudan University, 180 Fenglin Road, Shanghai, 200032 China; 2 Department of Geriatric Medicine, Affiliated Hospital of Nantong University, Nantong, Jiangsu China

**Keywords:** Mesenchymal stem cells, Heart failure, Stem cell transplantation

## Abstract

**Background:**

Heart failure (HF) is the end stage of most heart disease. Mesenchymal stem cells (MSCs), with their specific biological effects, have been applied in several clinical trials to evaluate the efficacy in HF therapy. We performed this meta-analysis to review the clinical evidence of their therapeutic effect on HF.

**Methods:**

Three databases were searched. The outcomes of interest were death, readmission, the 6-min walk test (6MWT), New York Heart Association (NYHA) class and left ventricular ejection fraction (LVEF). The relative risk (RR) and weighted mean difference (WMD) were calculated to evaluate the effects of MSCs on HF compared to placebo.

**Results:**

A total of nine studies were included, involving 612 patients who underwent MSCs or placebo treatment. The overall rate of death showed a trend of reduction of 36% (RR [CI] = 0.64 [0.35, 1.16], *p* = 0.143) in the MSC treatment group. The incidence of readmission was reduced by 34% (RR [CI] = 0.66 [0.51, 0.85], *p* = 0.001). The patients in the MSC treatment group realised an average of 40.44 m (WMD [95% CI] = 40.44 m [19.07, 61.82], *p* < 0.0001) improvement in 6MWT. The NYHA class was reduced obviously in the MSC group (WMD [95% CI] = − 0.42 [− 0.64, − 0.20], *p* < 0.0001). The changes of LVEF from baseline were significantly more than 5.25% (WMD [95% CI] = 5.25 [3.58, 6.92], *p* < 0.0001) in the MSCs group, unlike in the placebo group.

**Conclusions:**

Our results suggested that MSC treatment is an effective therapy for HF by improving the prognosis and exercise capacity. SCs derived from allosomes have superior therapeutic effects, and intracoronary injection is the optimum MSC delivery approach. Short-term cryopreservation is feasible in MSCs storage or transport.

## Introduction

Heart failure (HF) is a complex clinical syndrome resulting from structural or functional impairment of a ventricle [1]. Recent data shows that the prevalence of HF is approximately 1–2% among the adult population in developed countries, which constrains the quality of life and imposes a major societal burden [2]. Although various developing therapies, including non-invasive and invasive treatment, have increased the survival rate, patients with HF still experience high mortality and rates of hospitalisation [3]. Generally, non-ischemic HF and ischemic HF both result from cardiomyocytes dysfunction or death (e.g. necrocytosis, apoptosis), and unfortunately, cardiomyocytes cannot proliferate. Thus, an effective therapeutic strategy for myocardial restoration and/or regeneration may be one of the most significant fields in HF therapy.

Due to differentiation potential, stem cells have been profoundly studied for injured-tissue repair in clinical trials for more than 10 years [4]. Mesenchymal stem cells (MSCs) are fascinating for their unique cell phenotype. As a particular type of stem cell, MSCs not only have the self-replicating ability as well as the potential to differentiate into cardiomyocytes, but also have complicated biological effects, including but not limited to paracrine, anti-fibrosis and neovascularisation [5–8]. MSCs have been extensively studied, ranging from preclinical study to clinical study in recent years. Preclinical data, in both murine and large animal models, showed the benefits of MSCs by various mechanisms such as improving cardiac systolic function, promoting angiogenesis and alleviating ventricle remodelling [9]. Furthermore, multiple clinical studies with MSC treatment for heart disease have been conducted. In a recent meta-analysis, containing 58 preclinical studies and 6 clinical studies, has proved that the MSC therapy has benefits for patients with AMI (acute myocardial infarction) or ischemic cardiomyopathy (ICM) in a reduction in the infarct size and improvement of the left ventricular ejection fraction (LVEF) [10].

Based on the efficient biological effects, a number of clinical trials have been conducted to advance the application of MSC therapy for HF. The recent registry RCTs, TAC-HFT trial [5], MSC-HF [11], CHART-1 trial [12], and RIMECARD Trial [13] have been conducted to evaluate the efficacy in ischaemic HF and have been proved to improve left ventricular function and health status. The efficacy also was found in the randomised controlled trials (RCTs) among patients with non-ischaemic HF [13–17]. We performed this meta-analysis to review the clinical evidence for identifying the therapeutic effect on HF.

## Methods

### Strategy for literature search

To obtain available evidence, MEDLINE, Embase and the Cochrane Library were searched with the following terms “cardiac failure”, “myocardial failure”, “cardiomyopathy”, “myocardiopathy”, “heart decompensation”, “mesenchymal stem cell transplantation”, “mesenchymal stem cell”, “mesenchymal stromal cell” and “mesenchymal progenitor cell”. Furthermore, the reference lists from selected articles were double-checked for further relevant studies.

### Study selection

Studies were included according to the criteria as follows: (i) Adult patients diagnosed with HF by transthoracic echocardiography or cardiac magnetic resonance imaging, (ii) patients received MSCs or placebo treatment; (iii) study contained at least one of the clinical outcomes of death, readmission, six-minute walk test (6MWT), NYHA or LVEF and (iv) the follow-up duration was no shorter than 6 months. The exclusion criteria were as follows: (i) non-randomised controlled trial, (ii) insufficient data for statistical analysis, (iii) no full text of the original article and (iv) low-quality research.

### Data extraction

The study design, blind method, sample size and the information about the MSC therapy approach were collected to exhibit the characteristics of enrolled trails. We also extracted the baseline characteristics of the subjects in the studies, including gender, age, protopathy, New York Heart Association (NYHA) class and comorbidity. The primary clinical outcomes were death, readmission, 6MWT, NYHA and LVEF. Death was defined as the rate of all-cause death in our study. Readmission was defined as hospital admission due to decompensated HF. The changes of 6MWT and LVEF from baseline were calculated for comparison. Two investigators (Mengkang Fan and Yin Huang) searched the literature independently, and discussion was conducted with the third reviewer to solve disagreements. The authors of studies with insufficient outcome details were contacted to obtain missing data.

### Quality assessment

The quality of RCTs was evaluated using the Cochrane Collaboration tool to assess the risk of bias with the following terms: randomness of sequence generation, allocation concealment, blinding of participants and personnel, blinding of outcome assessment, incomplete outcome data, selective reporting and other potential sources of bias. Each item was classified as low risk, high risk or unclear, and the general risk of bias was determined by taking all items together and presenting it as a risk bias graph.

### Statistical analysis

Outcomes in our study, in the baseline characteristics of the enumeration data, are exhibited as *n* (ratio), and the measurement data is described as mean ± standard deviation. The dichotomous variables and the continuous variables were treated as relative risk (RR) [95% confidence interval (CI)] and weighted mean difference (WMD) [95% CI], respectively, in the meta-analysis. The fixed-effects model of the Mantel–Haenszel method or the inverse variance method was used to calculate the RR or WMD, respectively. Significant heterogeneity was considered when *p* ≤ 0.1 and *I*^2^ > 50%. The sources of heterogeneity were identified by sensitivity and subgroup meta-analysis. The subgroup meta-analyses were conducted with the donor, cell origin, approach of delivery, the preservation of cells and duration from acquisition to injection. The sensitivity analysis was estimated with a comparison between the results of combined effects, using the random-effects model and fixed-effects model. Furthermore, the sensitivity analysis was also performed using influence analysis to search for the studies that were responsible for heterogeneity when subgroup meta-analysis could not find the reasons for heterogeneity. Forest plots were used to present the results of the combined effect and the contribution of each study. Funnel plots were applied to visualise publication bias, and the Egger’s test was used for statistical assessment. Except for heterogeneity analysis, all *p* values were two-sided, and the significance level was set at 0.05. Stata/SE 12.0 was used to combine analyses and for the publication bias test.

This meta-analysis has been registered on PROSPERO (CRD42017079895) and was conducted in compliance with its recommendations [18].

## Results

### Eligible studies

**Fig. 1:**
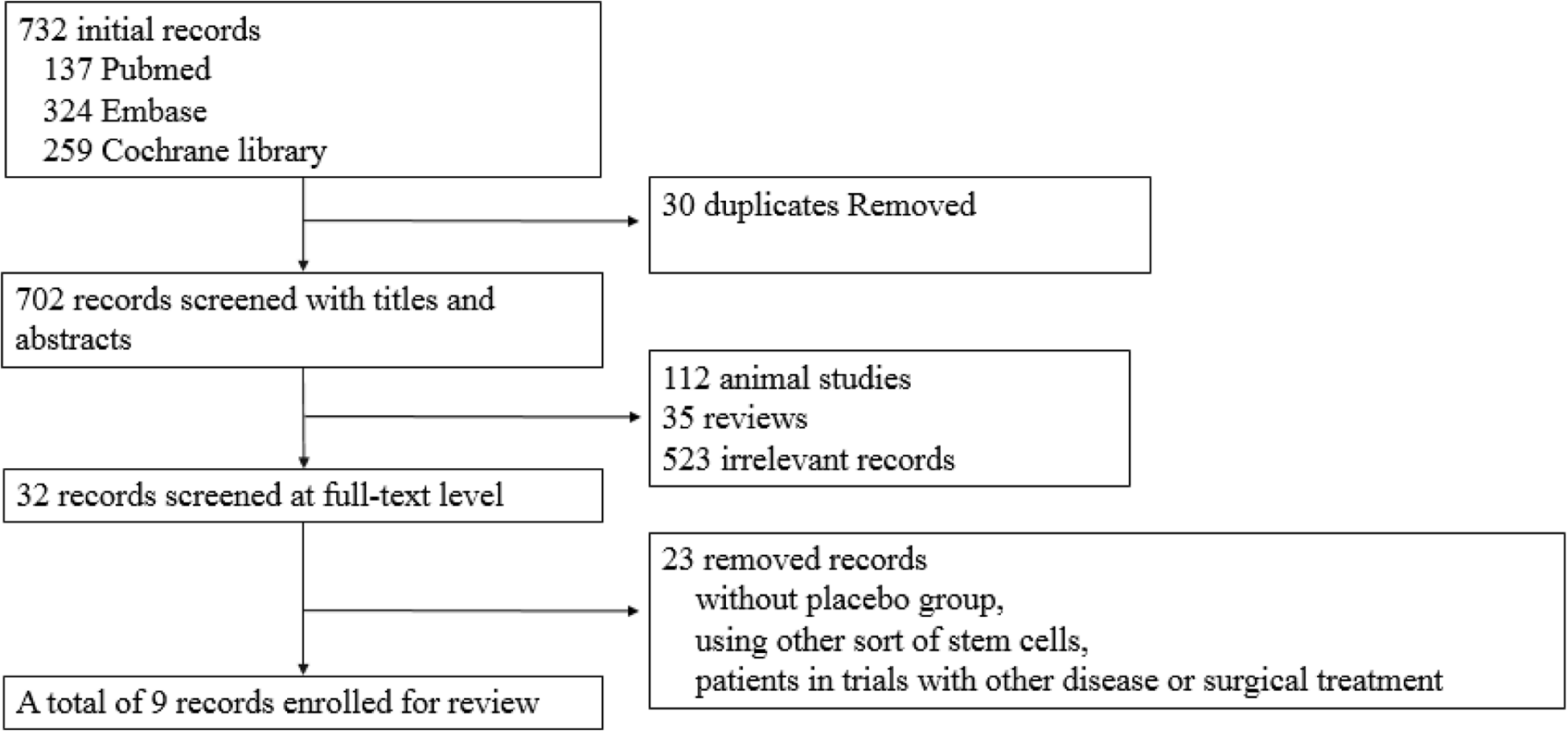
Flow chart of study selection

**Fig. 2:**
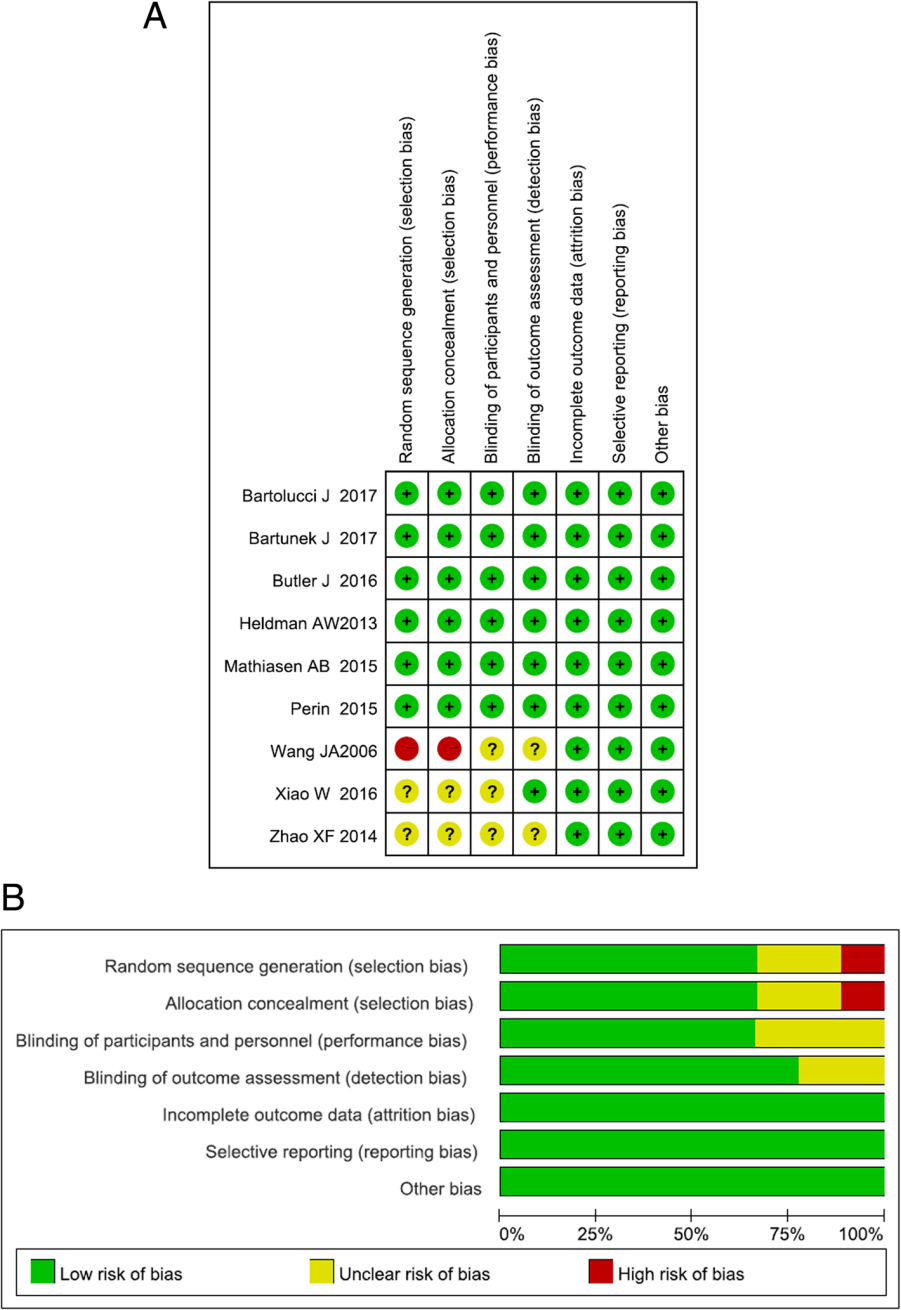
Risk of bias graph (**a**) and summary (**b**) of the included studies

The inclusion process of the studies for meta-analysis is shown in Fig. 1. By our search strategy, a total of 732 records were found (137 from MEDLINE and 324 from EMBASE), in brief, among which 35 were reviews, 702 were excluded after title and abstract screening, 32 were retrieved in full for detailed evaluation and 23 were excluded due to other cell types, primary disease and surgical treatment. Finally, nine studies [5, 11–17, 19] were included for further analysis. The Cochrane Collaboration tool was used to assess the risk of selection bias, performance bias, detection bias, attrition bias, reporting bias and other bias. The risk-of-bias graph is presented in Fig. 2.

### Characteristics of enrolled studies

As shown in Table 1, all nine studies were randomised controlled trials, four [5, 11–13] of which were double-blind trials; two [17, 19] were single-blind trials, and three [14–16] did not show the blind method. A total of 612 patients with HF were included, of which 263 and 304 underwent MSC and placebo treatment, respectively. Forty-five patients from one study [19], who underwent mesenchymal precursor cells (MPC) treatment, were also enrolled, because MPC shares many of the same phenotypic features as MSCs [20]. Among these studies, the origins of MSCs are varied: from autologous bone marrow in six studies [5, 11, 12, 14, 16, 17], from allogenic bone marrow in one study [19] and from allogenic umbilical cords in two studies [13, 15]. MSCs were delivered by intracoronary injection in three studies [14–16], by transendocardial stem cell injection (TESI) in four studies [5, 11, 12, 19] and by peripheral intravenous injection in two studies [13, 17]. The number of MSCs injected varied but in the same range of magnitude (10^6^~10^7^). Duration of follow-up was beyond 6 months in all nine studies. The characteristics of the patients in the included studies appear in Table 2. The majority of patients were male, and most of them were 40 to 70 years old. The protopathy was dilated cardiomyopathy (DCM) in two studies and ICM in two studies. In addition, patients with either DCM or ICM were included in three studies.

**Table 1:** Characteristics of included studies

Study	Date	Design	Blind method	Follow-up period	Sample size, *n*	Enrollment period	Cell origin	Delivery approach	MSCs dose	Placebo
Wang JA	2006	RCT	NA	6 months	24	2002.1–2004.10	Autologous bone marrow	Intracoronary injection	(5.86 ± 2.36) × 10^5^/ml × 30 ml	NS
Heldman AW	2013	RCT	Double-blind	12 months	30	NA	Autologous bone marrow	TESI	NA	Vehicle placebo
Zhao XF	2014	RCT	NA	6 months	59	2010.12–2011.12	Allogeneic Umbilical cord	Intracoronary injection	NA	NA
Mathiasen AB	2015	RCT	Double-blind	6 months	60	NA	Autologous bone marrow	TESI	(77.5 ± 67.9) × 10^^6^	PBS
Xiao W	2016	RCT	NA	12 months	37	2010.3–2011.6	Autologous bone marrow	Intracoronary injection	(4.9 ± 1.7) × 10^8^	NS
Butler J	2016	RCT	Single-blind	6 months	22	2014.6–2016.4	Autologous bone marrow	Intravenous injection	1 × 10^6^/kg	LRS
Perin	2015	RCT	Single-blind	12 months	60	2008.8–2010.6	Allogeneic bone marrow	TESI	25/50/75 × 10^6^	NA
Bartolucci J	2017	RCT	Double-blind	12 months	30	2012.12–2014.6	Allogeneic Umbilical cord	Intravenous injection	1 × 10^6^/kg	Autologous plasma
Bartunek J	2017	RCT	Double-blind	39 weeks	271	2012.12–2015.7	Autologous bone marrow	TESI	NA	NA

*RCT* randomised controlled trial, *TESI* transendocardial stem cell injection, *LRS* lactate Ringer solution, *NS* normal saline, *PBS* phosphate buffer saline, *NA* not available

**Table 2:** Baseline characteristics of the patients in the included studies

Study ID	*n* (MSCs/placebo)	Male, *n* (MSCs/placebo)	Age, years (MSCs/placebo)	Protopathy	NHYA class (MSCs/Placebo)	Hypertension, *n* (MSCs/placebo)	Diabetes, *n* (MSCs/placebo)	BMI (MSCs/placebo)
Wang JA	12/12	NA	NA	DCM	NA	NA	NA	NA
Heldman AW	19/11	18/10	57.1 ± 10.6/60.0 ± 12.0	ICM	2/2^*^	12/6	3/3	NA
Zhao XF	30/29	24/19	52.9 ± 16.32/53.21 ± 11.46	DCM and ICM	NA	NA	NA	NA
Mathiasen AB	40/20	36/14	66.1 ± 7.7/64.2 ± 10.6	ICM	29/15^*^	NA	15/3	29.8 ± 4.7/28.7 ± 5.3
Xiao W	17/20	12/14	51.6 ± 12.2/54.4 ± 11.6	DCM	NA	4/7	5/6	NA
Butler J	22	13	47.3 ± 12.8	NICM	1^*^	NA	5	NA
Perin	45/15	44/11	62.2 ± 10.3/62.7 ± 11.2	DCM and ICM	14/9^*^	29/9	13/2	29.8 ± 4.1/31.3 ± 9.2
Bartolucci J	15/15	12/14	57.33 ± 10.05/57.2 ± 11.64	DCM and ICM	2.03 ± 0.61/1.67 ± 0.49^†^	7/8	5/7	29.12 ± 2.88/29.52 ± 4.0
Bartunek J	120/151	107/136	61.6 ± 8.6/62.1 ± 8.7	NA	96/114^*^	NA	NA	28.2 ± 3.7/28.6 ± 4.4

*NHYA* New York Heart Association, *BMI* body mass index, *DCM* dilated cardiomyopathy, *ICM* ischemic cardiomyopathy, *NICM* non-ischemic cardiomyopathy, *NA* not available

*Indicate the number of patients with NHYA class III

### Clinical outcomes

#### Death

**Fig. 3:**
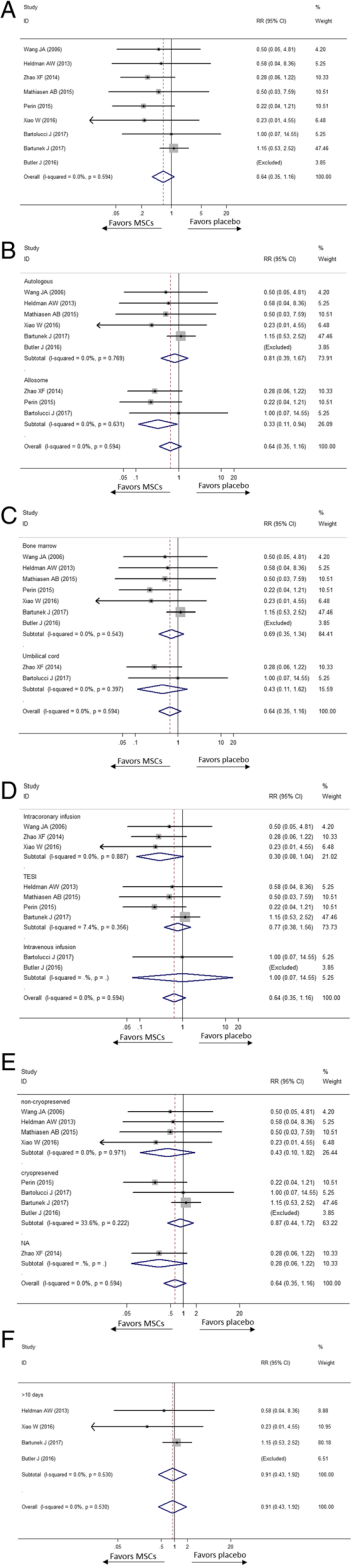
Effect of MSCs versus placebo on death: RR of death in the overall group (**a**), RR of death in subgroup of donor source (**b**), RR of death in subgroup of tissue origin (**c**), RR of death in subgroup of delivery approach (**d**), RR of death in subgroup of preservation (**e**) and RR of death in subgroup of duration over 10 days from acquisition to injection (**f**)

All nine studies were included for evaluation. As is revealed in Fig. 3, the overall rate of death in the MSC group exhibited a trend lower than in the placebo group by 36% (RR [CI] = 0.64 [0.35, 1.16], *p* = 0.143, *I*^2^ = 0.0%). In subgroup meta-analysis, mortality in the autologous MSC group and placebo group was similar (RR [CI] = 0.81 [0.39, 1.67], *p* = 0.564, *I*^2^ = 0.0%). However, the rate of all-cause death was significantly lower in the allogenic MSC group than in the placebo group (RR [CI] = 0.33[0.11, 0.94], *p* = 0.037, *I*^2^ = 0.0%). Moreover, no statistically significant mortality reduction was found in the bone marrow subgroup (RR [CI] = 0.69 [0.35, 1.34, *p* = 0.272, *I*^2^ = 0.0%) and umbilical cord-derived MSC group (RR [CI] = 0.43 [0.11, 1.62], *p* = 0.211, *I*^2^ = 0.0%). Although MSC transplantation by TESI did not significantly decrease the rate of death (RR [CI] = 0.77 [0.38, 1.56], *p* = 0.470, *I*^2^ = 7.4%), so did the subgroup of intravenous infusion group (RR [CI] = 1.00 [0.07, 14.55], *p* = 1.000), lower mortality among the MSC patients was detected in the intracoronary injection subgroup (RR [CI] = 0.30 [0.08, 1.04], *p* = 0.057, *I*^2^ = 0.0%). There were 4 of 9 studies cryopreserved MSCs in the vapour phase of liquid nitrogen before injection while 4 studies did not. In Fig. 3, the mortality was not reduced significantly neither by non-cryopreserved MSCs (RR [CI] = 0.43 [0.10, 1.82], *p* = 0.251, *I*^2^ = 0.0%) nor cryopreserved MSCs (RR [CI] = 0.87 [0.44, 1.72], *p* = 0.684, *I*^2^ = 33.6%). Among enrolled studies, 4 stated the duration from acquisition to injection which varied from 10 days to 5 weeks. The mortality in MSC group whose duration was beyond 10 days was similar with placebo (RR [CI] = 0.91 [0.43, 1.92], *p* = 0.806, *I*^2^ = 0.0%).

#### Readmission

**Fig. 4:**
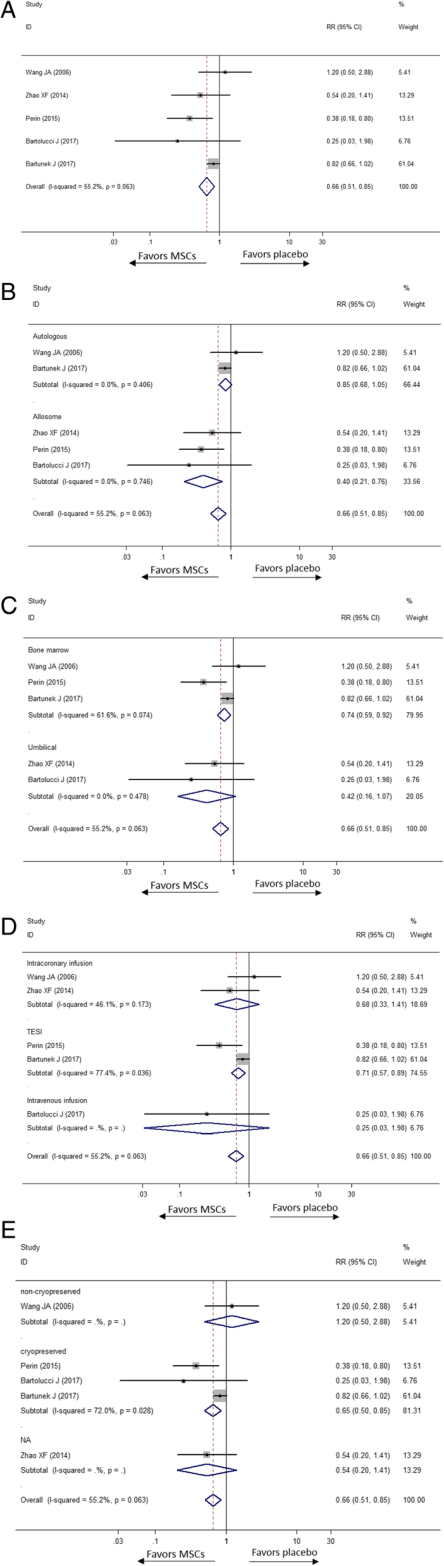
Effect of MSCs versus placebo on readmission: RR of the readmission (**a**), RR in subgroup of donor source (**b**), RR in subgroup of tissue origin (**c**) and RR in subgroup of delivery approach (**d**) and RR in subgroup of preservation (**e**)

Incidence of readmission for HF was observed in eight studies. According to Fig. 4, the overall incidence of readmission declined in the MSC group by34% (RR [CI] = 0.66 [0.51, 0.85], *p* = 0.001, *I*^2^ = 55.2%). In the subgroup analysis of cell origin, obvious reduction of incidence of readmission was detected in the allogenic MSC group (RR [CI] = 0.40 [0.21, 0.76], *p* = 0.005, *I*^2^ = 0.0%); meanwhile, in the autologous MSC group, the downtrend was not statistically significant (RR [CI] = 0.85 [0.68, 1.05], *p* = 0.122, *I*^2^ = 0.0%). Moreover, subgroup analysis of tissue recourse shows that the patients in subgroups who received MSCs from the bone marrow exhibited a lower incidence of readmission than the placebo group did (RR [CI] = 0.74 [0.59, 0.92], *p* = 0.006, *I*^2^ = 61.6%). However, there was no statistically significant difference between the umbilical MSC treatment group and the placebo group (RR [CI] = 0.42 [0.16, 1.07], *p* = 0.069, *I*^2^ = 0.0%). Furthermore, in subgroup meta-analysis of the delivery approach, the intracoronary and intravenous injection subgroups did not show a reduction of the incidence of readmission for HF significantly (RR [CI] = 0.68 [0.33, 1.41], *p* = 0.298, *I*^2^ = 0.0% and RR [CI] = 0.25 [0.03, 1.07], *p* = 0.190, respectively), and TESI presented a lower rate of readmission with obvious heterogeneity (RR [CI] = 0.71 [0.57, 0.89], *p* = 0.003, *I*^2^ = 77.4%). The rate of readmission was significantly reduced in the cryopreserved MSC group by 35% (RR [CI] = 0.65 [0.50, 0.85], *p* = 0.002, *I*^2^ = 72.0%), not in the non-cryopreserved MSC group (RR [CI] = 1.20 [0.50, 2.88], *p* = 0.684). Only one study reported both the readmission and duration from acquisition to injection, so we did not conduct this subgroup analysis.

#### 6MWT

**Fig. 5:**
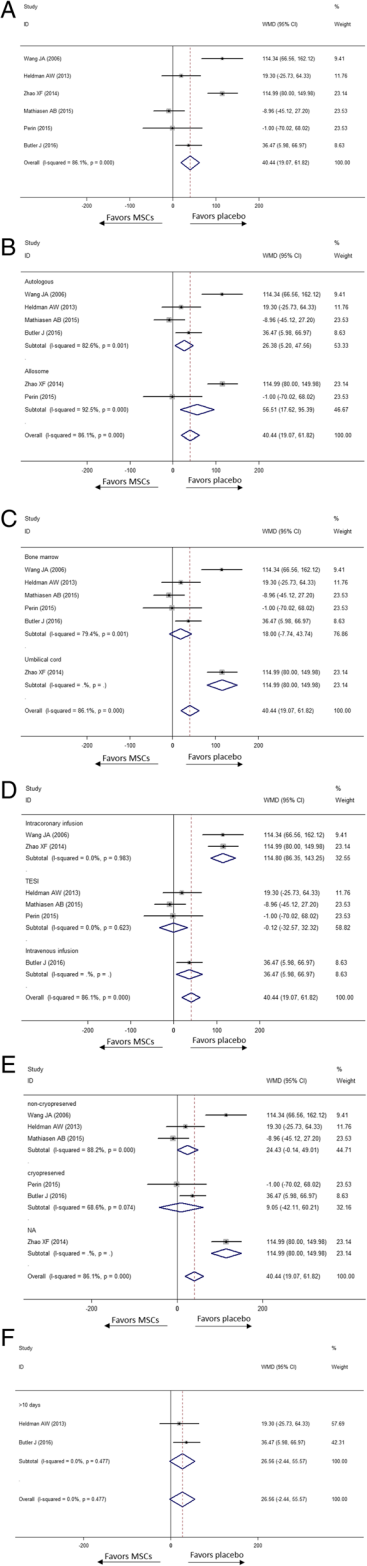
Effect of MSCs versus placebo on 6MWT: WMD of the change of 6MWT (**a**), WMD of 6MWT in subgroup of donor sources (**b**), WMD of 6MWT in the subgroup of tissue origins (**c**), WMD of 6MWT in the subgroup of delivery approaches (**d**), WMD of 6MWT in the subgroup of preservation (**e**) and WMD of 6MWT in the subgroup of duration over 10 days from acquisition to injection (**f**)

The 6MWT distance was measured in six studies before and after MSC treatment to evaluate the improvement of functional exercise capacity. As shown in Fig. 5, compared to the placebo group, the MSC treatment group increased 6MWT by 40.44 m with significant heterogeneity (WMD [95% CI] = 40.44 m [19.07, 61.82], *p* < 0.0001, *I*^2^ = 86.1%). Interestingly, in subgroup analysis, MSC therapy by TESI had no more benefit to increase functional exercise capacity than did placebo (WMD [95% CI] = − 0.12 m [− 32.57, 32.32], *p* = 0.994, *I*^2^ = 0.0%). Meanwhile, 6MWT significantly increased in the MSC injection by intracoronary and intravenous subgroups (WMD [95% CI] = 114.80 m [86.35, 143.25], *p* < 0.0001, *I*^2^ = 0.0% and WMD [95% CI] = 36.47 m [5.98, 66.97], *p* = 0.019, respectively). As shown in Fig. 5, the non-cryopreserved MSCs have a trend to improve the 6MWT by 24.43 m (WMD [95% CI] = 24.43 m [− 0.14, 49.01], *p* = 0.051, *I*^2^ = 88.2%) comparing with placebo. However, there was no obvious benefit shown in the cryopreserved MSC group (WMD [95% CI] = 9.05 m [− 42.11, 60.21], *p* = 0.729, *I*^2^ = 68.6%). In subgroup meta-analysis of duration from acquisition to injection, the 6MWT was increased by 26.56 m (WMD [95% CI] = 26.56 m [− 2.44, 55.57], *p* = 0.073, *I*^2^ = 0.0%) in the subgroup of duration over 10 days without significant heterogeneity.

#### NYHA class

**Fig. 6:**
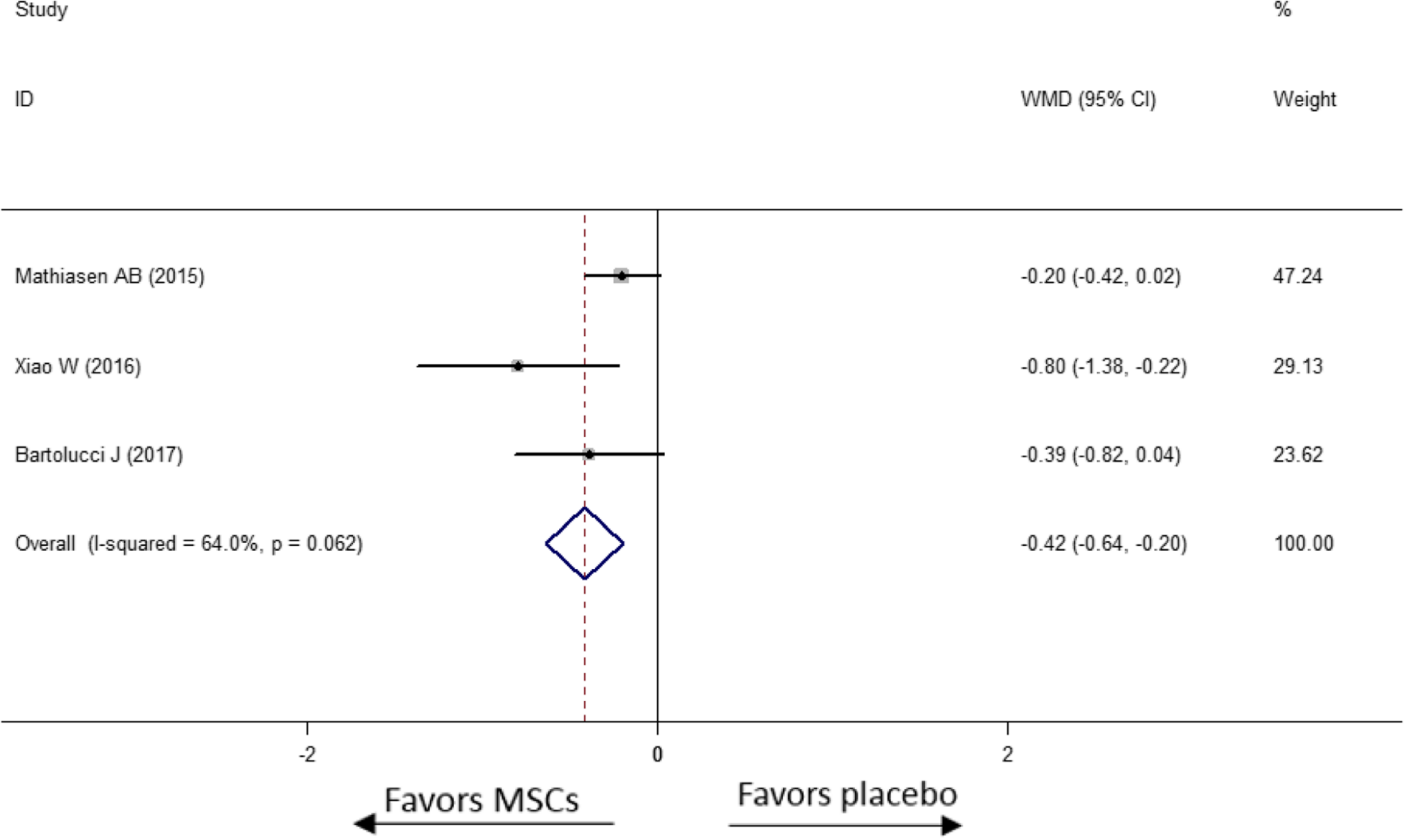
Effect of MSCs versus placebo on change of NYHA class

Though the data of NYHA grade was evaluated in 7 studies reported the baseline NYHA class, only 3 studies gave available data for meta-analysis. In Fig. 6, the NYHA class reduced more in the MSC group than the placebo group (WMD [95% CI] = − 0.42 [− 0.64, − 0.20], *p* < 0.0001, *I*^2^ = 64.0%) with moderate heterogeneity.

#### LVEF

**Fig. 7:**
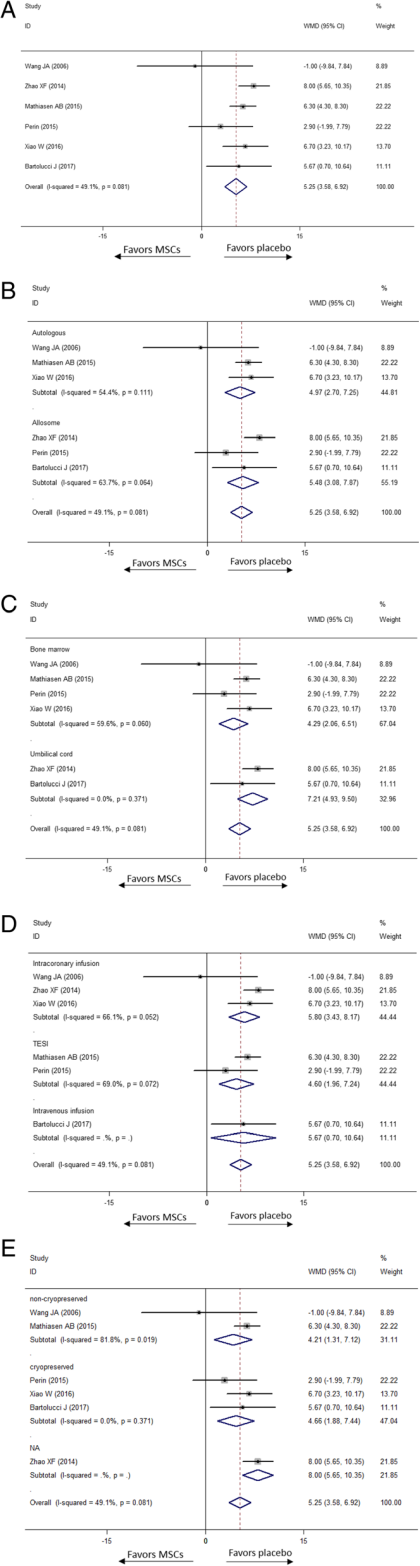
Effect of MSCs versus placebo on LVEF: overall WMD of LVEF (**a**), WMD in subgroup of donor source (**b**), WMD in subgroup of tissue origin (**c**), WMD in subgroup of delivery approach (**d**) and WMD in subgroup of preservation (**e**)

To assess left ventricular systolic function, LVEF was measured in eight studies, in which one tested LVEF by using magnetic resonance imaging (MRI), and the others by using transthoracic echocardiography. In Fig. 7, the overall variation of LVEF in the MSC group increased LVEF by 5.25% more than did the placebo group, with significant heterogeneity (WMD [95% CI] = 5.25 [3.58, 6.92], *p* < 0.0001, *I*^2^ = 49.1%). Moreover, a significant improvement of LVEF between these two groups was detected in all subgroup analyses. In the tissue-source subgroup, compared to placebo, patients who received treatment with the bone marrow and umbilical MSCs improved LVEF by 4.29% (WMD [95% CI] = 4.29 [2.06, 6.51], *p* < 0.0001, *I*^2^ = 59.6%) and 7.21% (WMD [95% CI] = 7.21 [4.93, 9.50], *p* < 0.0001, *I*^2^ = 0.0%), respectively. Similarly, in the cell-origin subgroup, LVEF could be increased by 4.97% (WMD [95% CI] = 4.97 [2.70, 7.25], *p* < 0.0001, *I*^2^ = 59.6%) and 5.48% (WMD [95% CI] = 5.48 [3.08, 7.87], *p* < 0.0001, *I*^2^ = 63.7%) than placebo, respectively, in the subgroups in which MSCs were derived from autologous and allogenic tissues. Statistical difference was also detected in the injection-approach subgroup. Intracoronary injection and TESI injection were proven to bring additional improvement of LVEF by 5.80% (WMD [95% CI] = 5.80 [3.48, 8.17], *p* < 0.0001, *I*^2^ = 66%) and 4.6% (WMD [95% CI] = 4.60 [1.96, 7.24], *p* = 0.001, *I*^2^ = 69.0%), respectively. One study, using peripheral intravenous injection to deliver MSCs, showed that MSCs increased LVEF 5.67% more than placebo (WMD [95% CI] = 5.67 [0.70, 10.64, *p* = 0.025]). Both MSCs non-cryopreserved and cryopreserved significantly improved LVEF more than placebo by 4.21% (WMD [95% CI] = 4.21 [1.31, 7.12], *p* = 0.004, *I*^2^ = 81.8%) and 4.66% (WMD [95% CI] = 4.66 [1.88, 7.44], *p* < 0.0001, *I*^2^ = 0.0%), respectively. Only one study reported both the LVEF and duration from acquisition to injection, so we did not conduct this subgroup analysis.

### Heterogeneity analysis

No heterogeneity was found by using the statistical method mentioned earlier in a meta-analysis of mortality. In addition, there was significant heterogeneity in meta-analysis of overall 6MWT. To search the source of the heterogeneity, we performed subgroup meta-analysis and found that heterogeneity no longer existed in the subgroup analysis of the transplantation approach. Although there was no statistic significant heterogeneity in the overall meta-analysis of readmission, heterogeneity still could not be ignored. Heterogeneity in the tissue-source subgroup and transplantation-approach subgroup was not found when combining the effects in each subgroup. For the analysis of LVEF, mild heterogeneity was noticed in the overall meta-analysis, although it did not have statistical significance. Meanwhile, subgroup meta-analyses did not ascertain the source of heterogeneity.

### Sensitivity analysis

**Fig. 8:**
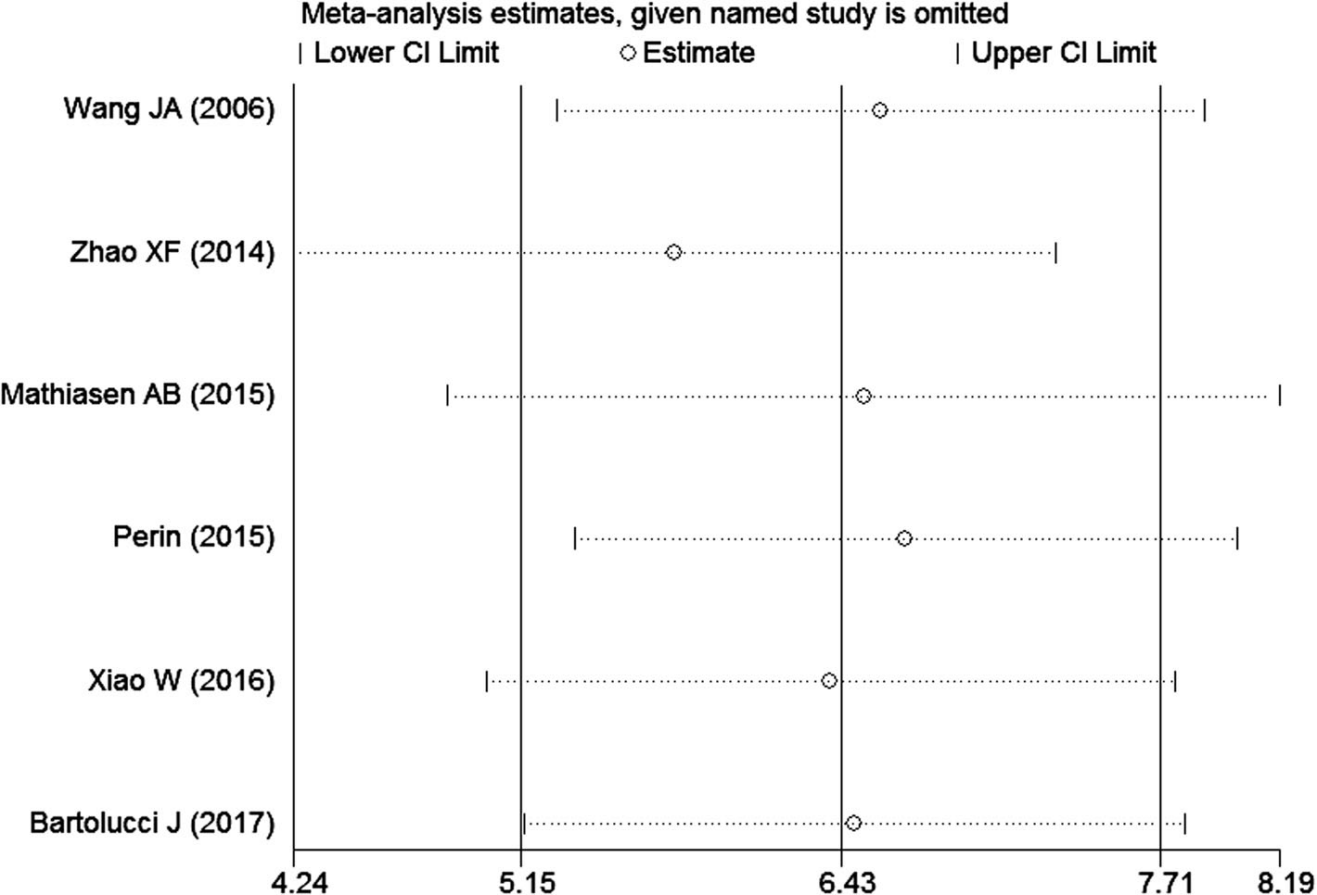
Influence analysis for changes in LVEF

The combined effects of death, readmission, 6MWT and change of LVEF, using the random-effects model, were similar to the fixed-effects model results. Moreover, because the source of the mild heterogeneity in changes in LVEF could not be identified by subgroup meta-analysis, influence analysis was necessary for a reliable explanation of the result of the meta-analysis. As we mentioned earlier, a sensitivity analysis was investigated by testing the influence of a single study on the overall meta-analysis for changes in LVEF. According to Fig. 8, Zhao XF’s study mostly influences the combined-effects value (WMD [95% CI] = 5.78 [4.24, 7.29]).

### Publication bias

As mentioned earlier, funnel plots were used to visualise the evaluation of the publication bias, and the Egger’s test was conducted for statistical assessment. The funnel plots exhibited approximately symmetric distribution (figures not shown). The Egger’s test showed that there was no significant publication bias in the meta-analysis for death (*p* = 0.125), readmission (*p* = 0.318), changes in 6MWT (*p* = 0.891) and changes in LVEF (*p* = 0.075).

## Discussion

The mean results of this meta-analysis are that MSC therapy might be able to reduce the survival rate and incidence of readmission, meanwhile improve the cardiac function among patients with HF. Importantly, the specific cell origin and delivery approach are essential for the strategy of MSC treatment. The cryopreserved MSCs benefited to reduce the rate of readmission and increased LVEF which indicated that cryopreservation is feasible for MSC storage. The duration from acquisition to injection longer than 10 days did not impact the therapeutic efficacy on mortality and 6MWT, and more data is required to reveal the roles in others’ end points in future.

In our meta-analysis, the overall rate of death was reduced by 36% in the MSC treatment group and the reduction was more significant, reaching70%, in the subgroup of intracoronary injection. MSCs from allosomes also reduced the mortality by 67%. The incidence of readmission was reduced by 34% and, in subgroups treated by allogenic MSCs, reduced by 60%. Compared to the placebo group, the autologous MSCs decreased the rate of readmission by 15% without statistical significance, which may be the reason for the heterogeneity. The improvement of functional exercise capacity was found in the MSC treatment group, with an average of a 40.44 m increase in 6MWT with significant heterogeneity. The heterogeneity vanished in the analysis of the delivery-approach subgroup. The group undergoing MSC treatment by intracoronary injection performed with a notably longer 6-min walk distance by 114.80 m on average, compared to the placebo group. On the contrary, the group that received MSC treatment by TESI showed no more benefit than the placebo group in 6MWT. The NYHA class is used in clinical practice more commonly to evaluate the exercise capacity. In our study, the NYHA class reduced more in patients treated with MSCs comparing with placebo. LVEF, as an acknowledged quantified factor for evaluation of left ventricular function, was a major clinical outcome in our study. The changes of LVEF from baseline were, significantly, 5.25% in the MSC group, more than in the placebo group. Taking all of the results together, MSC treatment showed benefits on mortality, the incidence of readmission, 6MWT and LVEF. Notably, even lower incidence of adverse events, and more improvement in cardiac function, were found in the allogenic MSC group and intracoronary injection group. The results demonstrate that MSC therapy offered a better prognosis for patients suffering from HF. Intracoronary MSC injection is the optimal transplantation approach, and MSCs derived from allosomes offer more benefits than autologous MSCs.

The MSC therapy in the heart has long been discussed. The safety and efficacy of MSC therapy for myocardial infarction (MI) have been confirmed by numerous clinical trials. Hare et al. performed a phase I randomised, double-blinded study in which acute MI patients were treated with allogeneic MSCs [21]. A significantly improved global symptom score, increased LVEF, reversed LV remodelling, improved pulmonary function and reduced ventricular arrhythmias were detected at 6 months after MSC injection. The APOLLO trial, a study of adipose-derived MSCs for ST-elevation MI, exhibited a trend of cardiac functional improvement, significant reduction in infarct size and an improvement in perfusion (ClinicalTrials.gov identifier NCT-01216995). Chen et al. [22] found that intracoronary autologous MSC injection for subacute MI improved LV chamber dimensions, ejection fraction and perfusion defects.

MSCs have been found in the human body and can be isolated from multiple organs including, but not limited to, the bone marrow, adipose tissue and umbilical cord. In the existed evidences, the MSCs isolated from either allogeneic or autologous tissues have been used in treatment. Each of the origins has advantages. The usage of autologous MSC can avoid potential ethical problems and ensure a reliable cell source. Meanwhile, the allogeneic MSC from healthy donors may easily to be standardised as an ideal “off-the-shelf” product. The efficacy of allogeneic MSCs in the restoration of cardiac function has been verified in various studies. In a preclinical research, the allogeneic MSCs engrafted in infarct and border zones and differentiated into cardiomyocytes, vascular smooth muscle and endothelial cell following transplantation into chronically scarred myocardium [23]. However, there were rare studies to compare the efficacy of autologous MSCs and the allogeneic MSCs. Our study suggested that allogeneic MSCs have greater efficacy than autologous MSCs in reducing mortality and incidence of readmission. Similar results were found in the POSEIDON-DCM trial, which was a randomised comparison of the safety and efficacy of autologous versus allogeneic bone marrow-derived MSCs in non-ischemic cardiomyopathy [24]. In the POSEIDON-DCM trial, allogenic MSC treatment showed significantly more improvement in 6MWT and the Minnesota Living with Heart Failure Questionnaire (MLHFQ), compared to autologous MSCs, and a lower rate of major adverse cardiac events (MACEs) and lower levels of tumour necrosis factor α (TNFα). In addition, allogeneic MSCs may activate more effective endogenous repair than autologous MSCs do [25]. Since HF is the end stage of the most heart diseases, the majority of the patients are the aged, so that the autologous MSCs therapeutic effects were impacted by the declined quantity and quality with age [26]. Thus, the potential reasons for the greater efficacy of allogeneic MSCs may be due to the difference of internal environments between patients and healthy donors [27]. The patients with HF commonly accompany with abnormal homeostasis, including, but not limited to, decline in regeneration, chronic inflammation and electrolyte disturbance. Because the current preclinical evidence is limited, further research is ongoing to accumulate solid evidence for a preferable MSC origin.

The MSC delivery approach in our study seems to be a key factor by which to determine the outcome of HF. Intracoronary MSC injection reduced the mortality and increased the 6MWT and significantly benefited for HF. The safety and efficacy of both approaches have been identified by a number of clinical trials. Controversially, Chin SP et al. [28] performed a study to investigate the efficacy of intramyocardial and intracoronary autologous bone marrow-derived mesenchymal stromal cell treatment for severe chronic dilated cardiomyopathy on LV parameters. The two injection approaches both improved the LV systolic function and alleviated LV remodelling at 12 months, compared to baseline. Although the differences of changes in LV parameters between those two approaches were not tested, a trend of more benefits from TESI was observed.

Inconsistency was also observed among preclinical studies. Perin et al. [29] found that TESI was superior to intracoronary delivery in a canine model of acute MI, validated by greater cell retention, increased vascularity and ideal functional improvement. On the other hand, Rigol et al. [30] used a porcine model of acute MI to compare two same-delivery routes and discovered that intracoronary injection increased neovascularisation compared to TESI, but these two methods had comparable engraftment rates. The biological effects of MSCs depend on several factors, such as the number of homing cells, activity and local microenvironment [9]. These complicated mechanisms may be the reasons for the conflicting evidence. In our studies, intracoronary MSC injection seemed to be the optimal method for the effects of MSCs in HF patients. Notably, a recent meta-analysis, containing preclinical and clinical studies, investigated the MSC treatment for acute myocardial infarction (AMI) which showed that the TESI seems to be a superior route of delivery [28]. In this research, the MSC infusion with TESI has a significant benefit to the end points, the infarct size and LVEF, while the MSC infusion with intracoronary injection did not. The potential reasons may be that even though coronary intervention performed timely when AMI happens, the myocardium hardly received complete reperfusion due to microcirculation dysfunction which may be a major threshold for MSCs [29]. Moreover, the acute ischemic region in ventricle has abnormal microenvironment which may active apoptosis of MSCs [30]. TESI can selectively deliver the MSCs to the non-infarcted region avoiding the obstacles in coronary artery microcirculation. However, in non-ischemic cardiomyopathy, the coronary perfusion was almost normal so that intracoronary injection may be more efficient. Besides, in chronic ICM, there was no coronary microembolisation caused by acute thrombosis and plaque rupture theoretically. In addition, the long-term ischaemia results in the improvement of coronary artery collateral circulation [31]. Furthermore, with the progress of ICM, the general remodelling and fibrotic substrate may adverse to the survival and functioning of MSCs in the myocardium which could limit the advantage of TESI. It indicated that the optimal route of delivery of MSCs for different heart disease may be various.

The preservation of the MSCs is a potential impact factor to its efficacy. Cryopreserved stem cells have been used in treatments for multiple diseases. Current evidences indicated that cryopreserved MSCs were able to maintain the expansion and differentiation ability. In a in vitro study, the cryopreserved MSCs have identical expression levels of characteristic markers of MSCs and had similar proliferation capacities [32]. Freezing of ex vivo-expanded MSC for 30 months did not affect the cell viability and ability [33]. A previous study has reported that cryopreserved MSCs were safe and feasible for the treatment of patients with severe dilated ischemic cardiomyopathy [34]. In line with the previous study, there was no obvious adverse effect on major end points in the present analysis. Besides, more than 10 days from acquisition to injection have no significant effect to the efficacy of MSCs in mortality and 6MWT. It suggests that short-term cryopreservation is feasible in MSC storage or transport which may facilitate the MSCs to be used as the on-shelf product.

A meta-analysis of the differences in changes of LVEF has shown that MSCs can provide an increased LVEF of 5.25% more than placebo; meanwhile, the source of heterogeneity cannot be detected by subgroup meta-analysis and sensitivity analysis. Although the heterogeneity is acceptable, it is necessary to be discreet when dealing with the results of the meta-analysis. First, the methods to evaluate the ventricular function and structure varied. Four studies measured these parameters by MRI, another four by echocardiography. Second, investigators in various medical centres may have different detection techniques. Hence, we speculate that these two reasons may be responsible for the heterogeneity.

## Limitations

This meta-analysis still has numerous limitations. First, the sample size in most of the studies was small, which may influence the results of the combined effect. Second, the pathogenesis of HF was not clearly classified in several studies. MSC homing and biologics may be affected by the microenvironment in the myocardium. The dysfunction of coronary artery microcirculation in ischemic cardiomyopathy may be a barrier against MSCs infused by the coronary artery. In addition, activation of the potential differentiation and external secretion of MSCs in a failing heart needs a specific microenvironment, which may be inappropriate, because the pathogenesis of non-ischemic cardiomyopathy is complicated and largely unknown. Thus, the diverse clinical efficacy of MSC therapy may lead to potential inconsistency among the studies. Last of all, most of the methods used to evaluate the quantification of exercise capacity and life quality were subjective, which restricts the persuasion of the data.

## Conclusion

According to our meta-analytic results from nine RCTs, MSC treatment is an effective therapy for HF by improving prognosis and quality of life. MSCs derived from allosomes have superior therapeutic effects, and intracoronary injection is the optimum MSC delivery approach. Moreover, short-term cryopreservation is feasible in MSC storage or transport. However, clinical trials with large sample sizes and standard procedures are urged to acquire more solid evidence for the application of MSCs.

## Abbreviations

6MWT: Six-minute walk test
CI: Confidence interval
DCM: Dilated cardiomyopathy
HF: Heart failure
ICM: Ischemic cardiomyopathy
LVEF: Left ventricular ejection fraction
MPC: Mesenchymal precursor cells
MSCs: Mesenchymal stem cells
NYHA: New York Heart Association
RCTs: Randomised controlled trials
RR: Relative risk
TESI: Transendocardial stem cell injection
WMD: Weighted mean difference
