# Correction to: Efficacy of mesenchymal stem cell therapy in systolic heart failure: a systematic review and meta-analysis

**DOI:** 10.1186/s13287-019-1329-3

**Published:** 2019-07-15

**Authors:** Mengkang Fan, Yin Huang, Zhangwei Chen, Yan Xia, Ao Chen, Danbo Lu, Yuan Wu, Ning Zhang, Juying Qian

**Affiliations:** 1 Department of Cardiology, Shanghai Institute of Cardiovascular Diseases, Zhongshan Hospital, Fudan University, 180 Fenglin Road, Shanghai, 200032 China; 2 Department of Geriatric Medicine, Affiliated Hospital of Nantong University, Nantong, Jiangsu China

**Correction to: Stem Cell Res Ther (2019) 10:150**

**https://doi.org/10.1186/s13287-019-1258-1**

The original article [1] displayed a co-author, Junbo Ge who has since stated that he should not have been a co-author in the article; the article has now been amended to remove Junbo Ge accordingly. All authors agreed to this amendment.

## References

[1] Fan M, Huang Y, Chen Z, Xia Y, Chen A, Lu D (2019). Efficacy of mesenchymal stem cell therapy in systolic heart failure: a systematic review and meta-analysis. BMC Med.

